# In Situ Synthesis Method of Approaching High Surface Capacity Sulfur and the Role of Cobalt Sulfide as Lithium–Sulfur Battery Materials

**DOI:** 10.1002/smsc.202300070

**Published:** 2023-08-17

**Authors:** Yew Von Lim, Sareh Vafakhah, Xue Liang Li, Zhuoling Jiang, Daliang Fang, Shaozhuan Huang, Ye Wang, Yee Sin Ang, Lay Kee Ang, Hui Ying Yang

**Affiliations:** ^1^ Pillar of Engineering Product Development Singapore University of Technology and Design 8 Somapah Road Singapore 487372 Singapore; ^2^ Pillar of Science Mathematics and Technology Singapore University of Technology and Design 8 Somapah Road Singapore 487372 Singapore; ^3^ School of Chemical and Biomolecular Engineering The University of Sydney Darlington NSW 2006 Australia; ^4^ Key Laboratory of Catalysis and Energy Materials Chemistry of Ministry of Education South-Central University of Nationalities Wuhan Hebei 430074 P. R. China; ^5^ Key Laboratory of Materials Physics Ministry of Education School of Physics and Microelectronics Zhengzhou University Zhengzhou 450052 P. R. China

**Keywords:** cobalt sulfide, CoS, lithium–sulfur batteries, polysulfide shuttling, sulfur batteries electrode design

## Abstract

Lithium–sulfur batteries (Li–S) are potentially applicable in electrification and the replacement of fossil fuels due to the high energy density and the economy of sulfur. However, effectively an insulator, sulfur is known to suffer from inert electrochemical and poor conductivity. By synthetically incorporating conductive, low‐dimensional carbonaceous composites acting as both containment hosts and catalysts to active materials, this work entails an effective and straightforward materials engineering approach in fundamentally remodeling active materials utilization and activation. This synthetic‐based approach highlights direct processing capabilities than traditional thermal infusion processes without compromising performance and addresses the low activation, poor conductivity as well as alleviating side reactions due to polysulfide species. Motivated by recent efforts in excellent catalytic properties of cobalt sulfide (CoS)‐based materials, in this work, high‐performance CoS‐based carbonaceous composites are designed and employed, alleviating side reactions. These sulfide‐based catalysts are further elucidated in their role in facilitating charge/discharge of active materials, and assessed on practical polysulfide and side reaction alleviation with respect to various discharge/charge states. Proof‐of‐concept devices demonstrate the following performance highlights: 1) high‐performance stability, 2) strong polysulfide adsorption capability and kinetic characteristics, 3) large and workable areal capacity, and active materials loading.

## Graphical abstract

A direct synthetic‐based in situ approach is developed to utilize low‐dimensional‐based carbonaceous composites, specifically that of reduced graphene oxide–carbon nanotubes (rGO–CNT), as active materials repository and catalytic materials support. The conductive enhancement involving the incorporation of carbonaceous composites is evident in the performance improvement of the lithium–sulfur batteries.

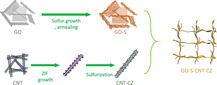

## 1 Introduction

There is a strong demand for lithium‐based energy storage devices mainly attributed to the worldwide push to adapt to sustainable energy resources and renewables.^[^ ^1^, ^2^, ^3^ ^]^ As such, it is expected that novel, postlithium storage technologies that are high performance and more economical should continue to be the focal point for both the industry and research community. The key to the acceptance of this diversification is mainly driven by the economic aspect spanning from research cost, supply chain, and ultimately in‐process manufacturing.^[^ ^4^ ^]^ Slotting perfectly in all of these aspects, lithium–sulfur batteries (Li–S) emerge as highly attractive ascribed to their excellent energy density (≈600–2600 Wh kg^−1^, >10 times of current technologies), and the natural abundances of sulfur.^[^ ^1^, ^2^ ^]^ However, at the current stage, it is crucial to realize practical storage potentials of the storage materials by addressing sulfur's electrochemical and conductive inertness. This can only be approached by re‐engineer how sulfur is activated from the fundamental perspective of materials: 1) Bulk sulfur cannot be utilized readily as active materials without treatment. The lack of electrochemical activity as functional storage materials needs to be addressed starting from the micro/nanoscale perspective and is beyond systematic level.^[^ ^5^, ^6^, ^7^ ^]^ 2) Recent efforts established that it is effective to uniformly disperse/distribute active materials both as composite electrode materials or in layered design (functional layers or as separators).^[^ ^6^, ^8^, ^9^ ^]^ 3) The conductivity of active materials can be enhanced by incorporating conductive materials and is one the key attributes to activation.^[^ ^10^, ^11^ ^]^ 4) Catalytic treatment targeting on discharged polysulfide (LPS) intermediates is necessary due to the dissolution of these intermediates ultimately at the electrolyte and anode of the cell resulting in performance fading, a commonly encountered side reaction known as polysulfide shuttling.^[^ ^12^, ^13^, ^14^ ^]^ As of current, it is clear that the prospects of the technology remained, and is crucial, in developing materials and constructs that are adaptable and high performance. As such, this underlines the major hurdles in affecting practical realizations of Li–S and is equally critical on other similar storage technologies such as that of sodium and potassium sulfur batteries.

Recent efforts on addressing these issues mostly emphasized on the catalytically attributed improvements and the elucidations of electrocatalysts in the role in performance enhancement, i.e., to elicit adsorption of redox intermediates (by using polar molecules), and facilitating LPS conversion.^[^ ^12^, ^15^ ^]^ Impressive progresses focusing on achieving high‐performance electrocatalysts design and the study via electrochemical assessments with theoretical evaluations, were reported. As a direct approach in alleviating conductive inadequacies and intermediates facilitation, utilizing highly conductive host material with the incorporation of strong LPS adsorption electrocatalyst is proven to be effective.^[^ ^16^, ^17^, ^18^ ^]^ In one of our contributions, we revealed the efficacy of phosphide‐based electrocatalyst (iron phosphide, FeP) and its incorporation to highly conductive low‐dimensional carbonaceous composites in addressing conductive inertness of sulfur.^[^ ^16^ ^]^ FeP exhibits strong LPS adsorption capability contributing to high‐rate performance and is the key in alleviation of LPS shuttling. Another important and recent work is reported by Zhou et al., in which systematic assessments on catalytic activities were conducted on a series of transition metal sulfides (VS_2_, CoS_2_, TiS_2_, FeS, SnS_2_, and Ni_2_S_3_).^[^ ^19^ ^]^ The studies involved evaluation and correlation of important parameters such as LPS adsorption capability to the redox energy of the catalytic materials. In comparison to various transition metal‐based compounds such as phosphides^[^ ^7^, ^16^, ^20^, ^21^ ^]^ and oxides,^[^ ^22^, ^23^, ^24^ ^]^ metal sulfides^[^ ^25^ ^]^ remain as one of the more widely trialed materials due to high surface reactivity, electric conductivity, and materials stability.^[^ ^26^, ^27^, ^28^ ^]^ In practical sense, however, it must be noted that the loading of sulfur for these stated approaches is normally achieved via thermal infusion and requires additional processes involving the use of high temperature, within vacuum environment and long processing durations. In terms of addressing the efficacy of electrocatalysts and host materials design, such impregnation approaches are often necessary due to the larger surface area of contact which the electrocatalysts are designed to target and thus can only work with sublimated and vaporized sulfur (to effectively incorporate these materials constructs to sulfur in a uniform manner).^[^ ^29^, ^30^ ^]^ It is also worthwhile to mention that other simpler, yet less subtle impregnation methods, may result in further complications of affecting the overall morphology and structure of host‐catalyst composite. Therefore, the overall expected performance efficacy of the construct should be affected. For a significant number of these works, the results of using thermal infusion usually attribute to usable but a rather low active materials loading (<50% w/w).^[^ ^31^, ^32^, ^33^ ^]^ Even with excellent device performance attributes (C‐rate and performance stability), it is preferable to have a high active materials loading, which can actively encourage practical adaptation and utilization. As of today's requirement, it becomes progressively evident that along with the high‐performance attributes, the acceptance of the technology should require potential realizable emphasis in the aspect of processing efficiency, feasibility, and prospect for systematic upscaling and improvement.

As such, we sought to resolve the above starting from the perspective of how the active material should be effectively approached with practical prospects without compromising resulted in performance. A direct synthetic‐based in situ approach is developed to utilize low‐dimensional‐based carbonaceous composites specifically that of reduced graphene oxide–carbon nanotubes (rGO–CNT), as active materials repository and catalytic materials support. Different from the conventional host design in which the active materials are incorporated via vapor infusion, we successfully established in incorporating active and catalytic materials synthetically. The conductive enhancement involving the incorporation of carbonaceous composites is evident in the low‐impedance profiles and is vital in contributing to the performance improvement. Another important highlight of this work is that the zeolitic imidazolate framework (ZIF)‐derived, sulfide‐based catalysts (cobalt sulfide, CoS) were evaluated and assessed, an important milestone for this very effective class yet rarely reported sulfide‐based catalyst. In recent efforts, studies on the use of cobalt sulfides have shown excellent performance when employed as electrocatalysts for water splitting, oxygen evolution reaction, hydrogen evolution reaction, etc.^[^ ^34^, ^35^, ^36^, ^37^, ^38^, ^39^, ^40^ ^]^ Hu et al. revealed that apart from the unique chemical composition, the interaction effect of S‐doping atoms on carbonaceous materials, especially for CoS, strongly attributes exceptional electrocatalytic activity.^[^ ^41^ ^]^ Chen et al. also commented that sulfide has higher catalytic activity due to the S(‐II) low valence in facilitating cycle of metallic valence.^[^ ^42^ ^]^ Moreover, various efforts further reveal that its excellent electron/charge transfer properties, narrow bandgap, and efficient catalytic activation endow CoS with excellent catalytic capability.^[^ ^43^, ^44^, ^45^ ^]^ In overall, in our effort, excellent performance and utilization attributes (high rate at 2.0 C and cycling stability at >1800 cycles; large areal capacity and performance stability 6.5–8.1 mAh cm^−2^) are achieved.

## 2 Results and Discussion

**Scheme 1:**
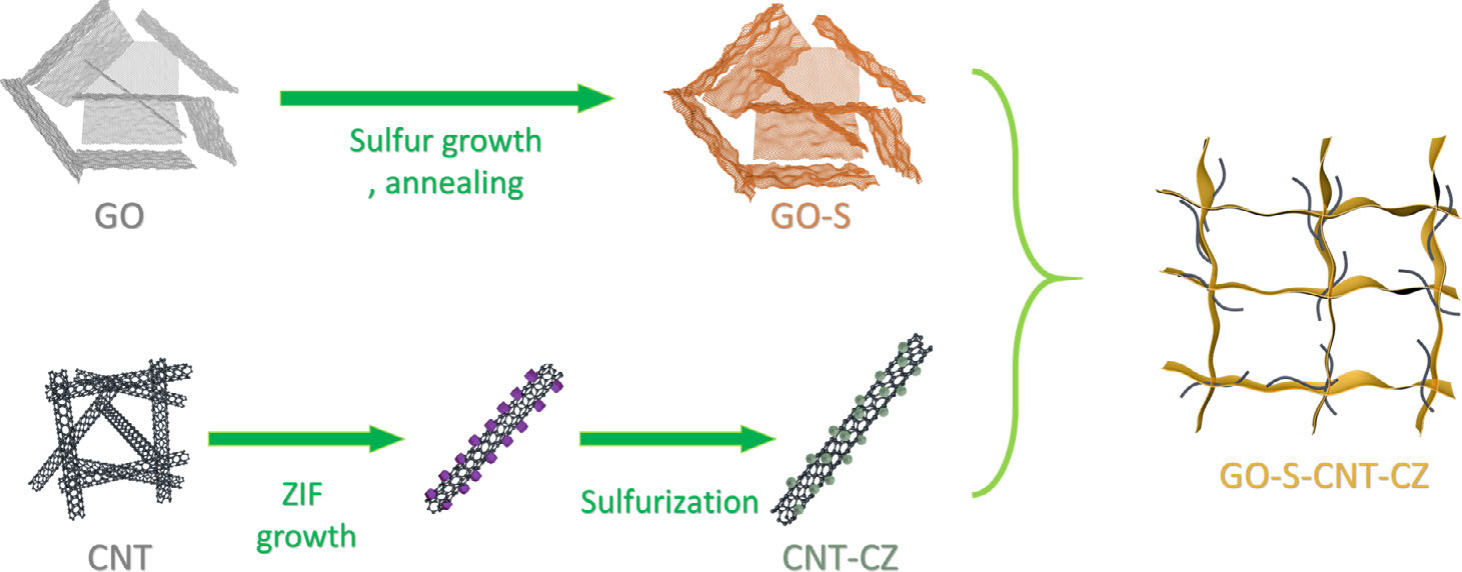
Synthesis process of active material host (GO‐S), ZIF‐derived catalytic material host (CNT‐CZ), and the formation of the composite GO‐S‐CNT‐CZ via lyophilization.

**Figure 1:**
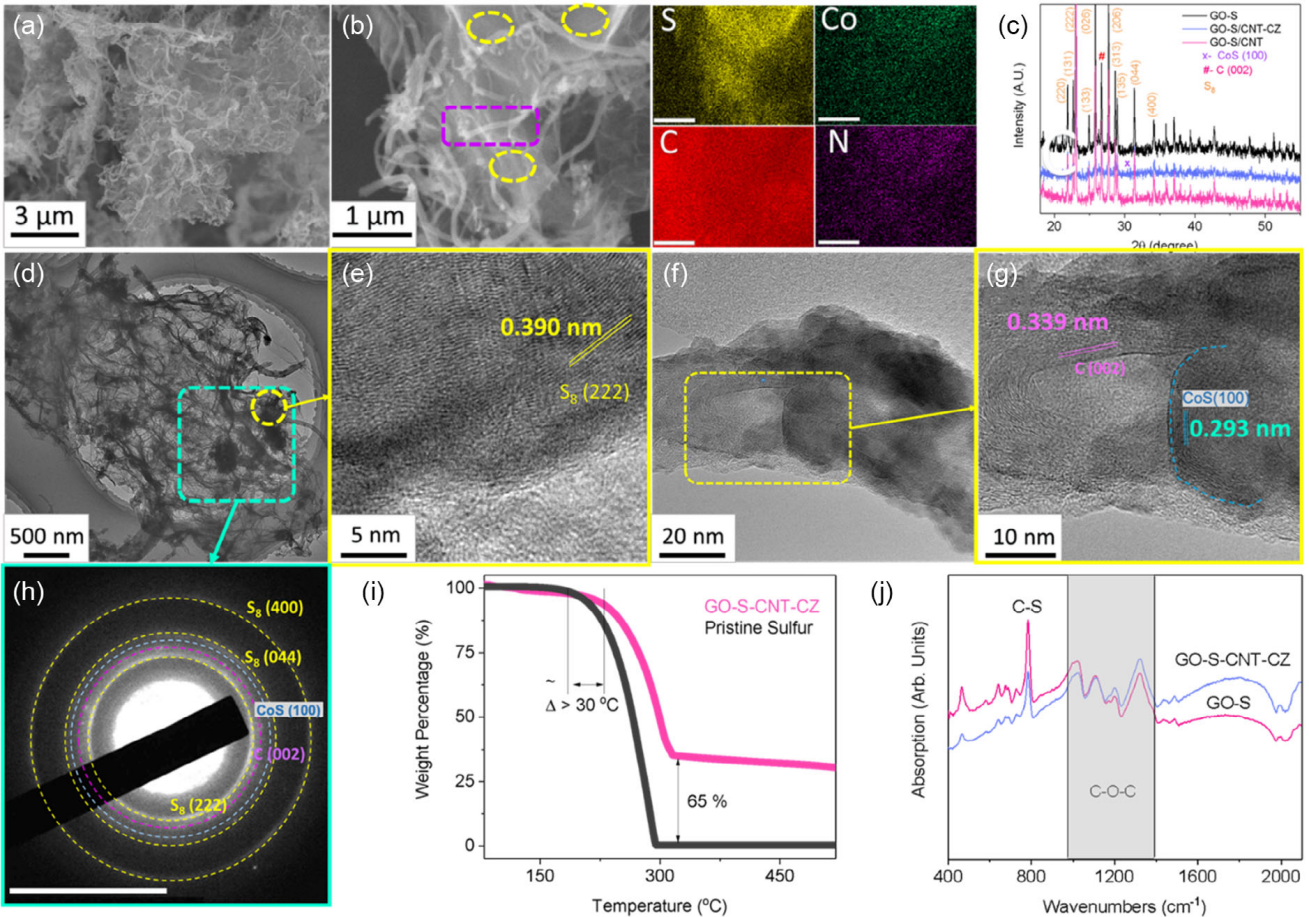
Materials characterization results of the GO‐S‐CNT‐CZ and its precursors. a) SEM result. b) SEM of a magnified region of GO‐S‐CNT‐CZ and its corresponding EDS mapping image. c) XRD pattern in comparison with various samples. d) HR‐TEM image. e) Magnified TEM image of a region marked in (d). f) HR‐TEM image of the CNT‐CZ precursor. g) Magnified TEM image of the region marked in (f). h) SAED pattern of GO‐S‐CNT‐CZ at a region marked in (d). i) TGA profiles. j) FTIR spectra.

The synthetic incorporation of the active sulfur, the formation of the ZIF‐derived catalysts, and the incorporation to achieve the growth of carbonaceous host with catalytic‐supported active materials are shown in (**Scheme** **1**). First, graphene oxide solution (GO) was incorporated with sodium persulfate as synthetic precursors to prepare sulfur growth in GO. As a result of abundance of grain boundaries and functionalized groups on GO, sulfur was synthesized readily via thermal activations to these locations. Serving as synthetic sites, these grain boundaries sites exhibit uniform growth of sulfur, and which the sulfur was branching out from these locations as indicated in energy‐dispersive spectroscopy (EDS) and related scanning electron microscopy (SEM) results (Figure S1, Supporting Information). The thermal‐based reduction process is necessary to further reduce GO (Figure S2, Supporting Information) to reduced graphene oxide (rGO, **Figure** **1**c) acting both as uniform sulfur repository and conductive support. In a separate synthesis, ZIF nanoparticles were incorporated with carbon nanotubes (CNT) by sonication. The resultant CNT‐ZIF composite was then pyrolyzed/sulfurized in which the resultant daisy‐chained like CNT/CoS (CNT‐CZ) composite was achieved (Figure S3, Supporting Information). EDS results of CNT‐CZ correspond to strong C, Co, and S signals. Co and S indicate close to 1:1 elemental/atomic ratio and almost 8.0 at% with respect to the overall construct. As a subsequent process in combining both composites, both CNT‐CZ and rGO@sulfur composites were rapidly frozen in liquid nitrogen environment from room temperature, resulting in the rapid volumetric reduction of rGO@sulfur (GO‐S) and the incorporation of the two precursors. The unique morphology of the composite construct and the success of the incorporation is motivated by the shock freezing/rapid thermal cooling in driving: 1) graphene host materials to shrink, and 2) CNT and the graphene host combining at their functional sites resulting from the rapid freezing effect. In overall, the morphology of the composites demonstrates successful incorporation of rGO‐based active material host and that of the CNT‐ZIF catalytic support as GO‐S‐CNT‐CZ (Figure 1a,b). XRD results (Figure 1c) indicate that the ZIF‐based nanostructure is successfully sulfurized to CoS, by the formation of CoS (100) (PDF‐00‐075‐0605).^[^ ^46^ ^]^ The same XRD pattern is not observed when contrasting to GO‐S and GO‐S incorporated with pristine CNT (GO‐S‐CNT). Therefore, we attribute the formation of the CoS (100) reflection patterns to that of the formation of the sulfurized precursor. In contrast (Figure 1c), highly intensified XRD patterns are observed in all composite samples and are referred to as molecular sulfur (S_8_, PDF‐00‐024‐0733). To confirm the formation of the CoS in GO‐S‐CNT‐CZ, we also subject CNT catalyst host of various nature (CNT‐CZ before and after sulfurization, and pristine CNT) to XRD investigation (Figure S4, Supporting Information). The result indicates that the weak XRD pattern ascribed to CoS (100) reflection is observed and only limited to the CNT‐CZ composite, highly indicative on the formation of CoS structure. The results also ruled out that the weak XRD pattern is due to the sulfur precursors. In‐depth morphology was further examined by high‐resolution transmission electron microscopy (HR‐TEM) analysis. HR‐TEM image indicates the same morphology as shown in the SEM results (Figure 1d). The grain boundaries region of the rGO is much darker in comparison to other regions due to sulfur growth and is identified via the measured lattice interlayer spacing and corresponding diffraction profiles (Figure 1e). Specific focus was dedicated on the CNT‐CZ regions (Figure 1f), in which nanoclusters of CoS were identified^[^ ^46^, ^47^ ^]^ and in the region of 15–20 nm in diameter and are uniformly grown on the surface of CNT (Figure 1g). The selected area diffraction (SAED, Figure 1h) pattern was taken and identified the chemical stoichiometry and formation of CoS (100) and confirmed on the formation of CoS. We have also provided another direct investigation via Raman analysis as a convincing proof for the formation of CoS (Figure S5, Supporting Information). The *E*_g_ and the *A*_1g_ band of CoS^[^ ^48^ ^]^ were observed indicating the formation of the CoS structure, supporting on the previous observation on the success in formation of the CoS structure. Quantification on active sulfur of the composite was assessed by thermogravimetric analysis (TGA) and in reference to bulk sulfur (Figure 1i). The overall active materials loading was evaluated as 67% w/w. It is worthwhile to note that the thermal decomposition profile of the composites differs from that of pure sulfur. For the composites, although the sulfur grains can mostly be identified in XRD results and are similar to the large molecular sulfur S_8_, the binding toward rGO due to the synthetic nature is able to prolong the thermal decomposition of sulfur species by more than 30 °C. The nature of the thermal stability was further assessed and confirmed by Fourier‐transformed infrared spectroscopy (FTIR). From the formation of the strong C—S bond (780–800 cm^−1^) as shown in the FTIR spectra (Figure 1j),^[^ ^48^, ^49^ ^]^ we established that the synthetic nature of the active materials results in the chemical‐bonded characteristic of the carbonaceous host to the synthetically‐derived sulfur. The chemically bonded nature of the active materials is highly suggestive toward high thermal stability and mechanical stability due to the bonding with the graphene species. As a result, we expect these vital findings to be advantageous in contributing to electrochemical performance as detailed in later sections.

**Figure 2:**
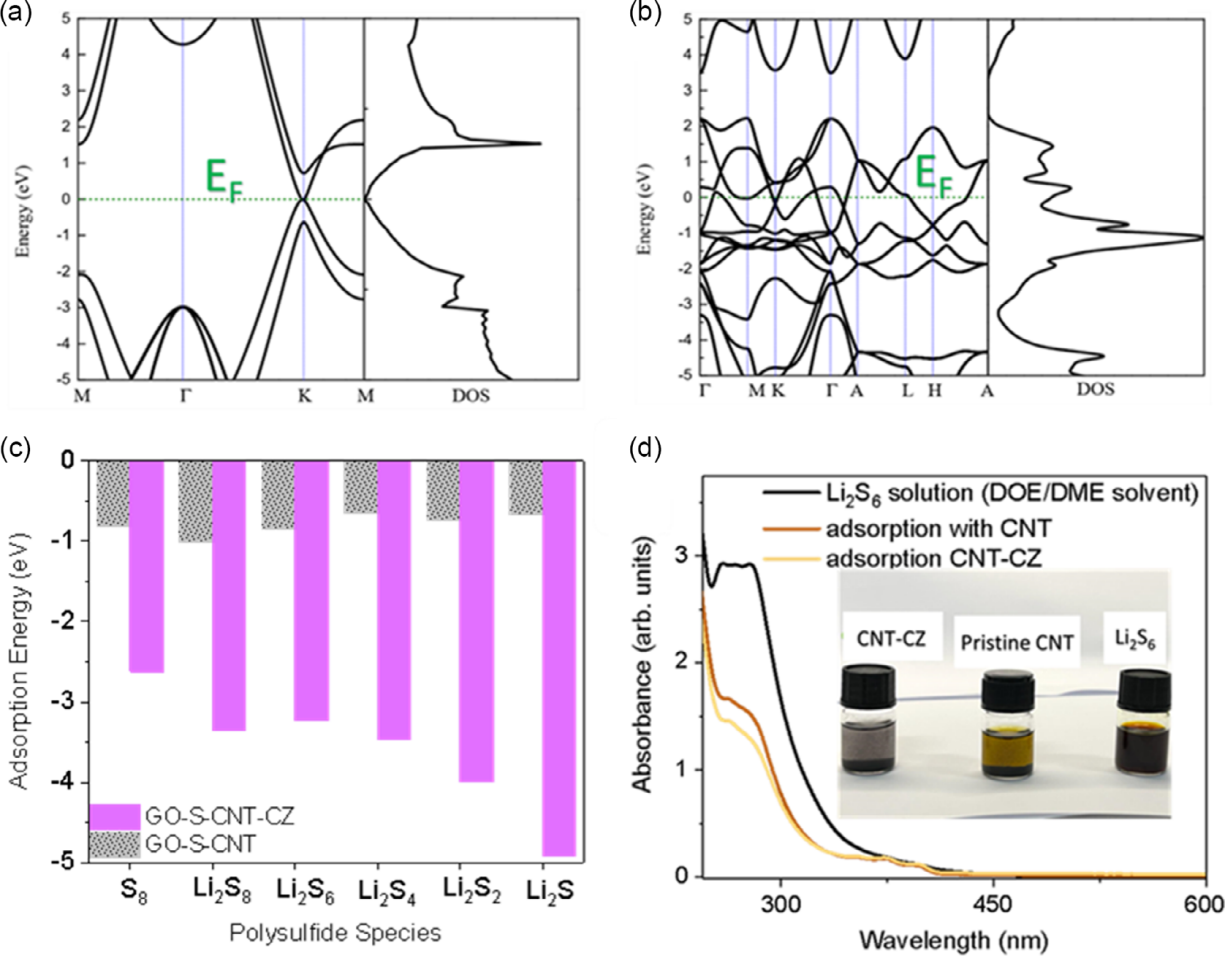
a,b) Band structure with the density of states (DOS) of CNT (a), CoS (cobalt sulfide) (b). c) Adsorption energy of CNT‐CZ and pristine CNT toward sulfur and LPSs species. d) UV–vis spectra of the polysulfide solution after the adsorption test, inset indicates the respective solutions under investigation (the Li_2_S_6_ processed under CNT‐CZ, pure CNT, and the Li_2_S_6_ as prepared).

We further evaluated and subjected CoS to first‐principles calculation in assessing its overall electrochemical catalytic behavior to the active materials and the redox intermediates. First, the band structure of CoS@carbonaceous materials (model and representing CNT‐CZ) is evaluated and compared to graphitic carbon (model and representing pristine CNT without CoS). The Fermi energy (*E*_F_) is set and normalized to the origin. The results revealed that the density of states (DOS) is more abundant (**Figure** **2**a) around the *E*_F_ in contrast to that of pure CNT, a testament of lower energy inclination of charge transfer for the CNT‐CZ versus pristine CNT. The band structure also reveals that CoS has almost negligible bandgap and indicates the semimetallic behavior and is energetically favorable for charge transfer and diffusion. The adsorption behavior toward redox intermediates (i.e., LPS species and active materials) is one of the vital components in the efficacy of the catalyst. Density functional theory (DFT) calculations reveal that adsorption energies of the CNT‐CZ corresponding to the redox products of S_8_ to Li_2_S, are substantially more exothermic in stark contrast to CNT (Figure 2c). Overall, both species exhibit strong adsorption capability toward the redox intermediates (i.e., negative and stabilized energies).

To further demonstrate polysulfide adsorption ability of the CoS catalyst, the highly porous CNT‐CZ composite was subjected to LPS adsorption test. We first analyzed the composition of CoS within the CNT‐CZ composite via TGA (Figure S6, Supporting Information). The profile exhibits first a downward trend (≈10%, <150 °C) in weight lost due to stripping of moisture. Subsequently, there is another gradual drop in weight profile from ≈250 °C, and is ascribed to the removal of sulfur as previously reported.^[^ ^50^ ^]^ The major weight lost in the composite commences at 520 °C due to the loss of graphitic carbon from the CNT.^[^ ^51^ ^]^ In overall, the composition of the standard composite CNT‐CZ is evaluated as 65% w/w of C and 35% w/w CoS. This attribute to about 7.3 at% of CoS and is consistent to the EDS findings (indicated at 8.0 at%). The adsorption capability of the composite was investigated via the interaction of the catalytic composite with synthetic Li_2_S_6_ in 1,3‐dioxolane/1,2‐dimethoxyethane (DOL/DME) solution (4 mL, 10 mm) and aged for 360 min without agitation. The standard CNT‐CZ composite was compared with pristine CNT in this process and which the former displayed drastic color fading ascribed to the interaction of the LPS to the highly adsorptive CoS (inset of Figure 2e). In comparison, pristine CNT shows lesser adsorption capability toward LPS with very subtle color change. UV visible spectroscopy was deployed to quantify the visual findings (Figure 2d). The absorbance contribution of Li_2_S_6_ at 260–300 nm is quenched more drastically than the control sample.^[^ ^17^ ^]^ The absorbance contribution at 260–300 nm shows suppressed intensity from the slopping background thus indicates that the efficiency of LPS adsorption and facilitation capability of the CoS electrocatalysts in effectively capture/adsorb the LPS species. We also subjected CNT‐CZ catalytic host precursor to further morphological quantification. N_2_ sorption isotherm results via Brunauer–Emmett–Teller (BET) analysis (Figure S7, Supporting Information) show that the ZIF‐derived sulfide‐based catalyst contributes to excellent specific surface area (128.558 m^3^g^−1^). The considerably large surface area is crucial for processing and adsorption of the redox LPS during charge/discharge. It is thus evident that these findings verify the excellent remediation efficacy of CoS and thus signify the excellent prospects of the sulfide‐based catalytic material in nullifying LPS side reactions.

**Figure 3:**
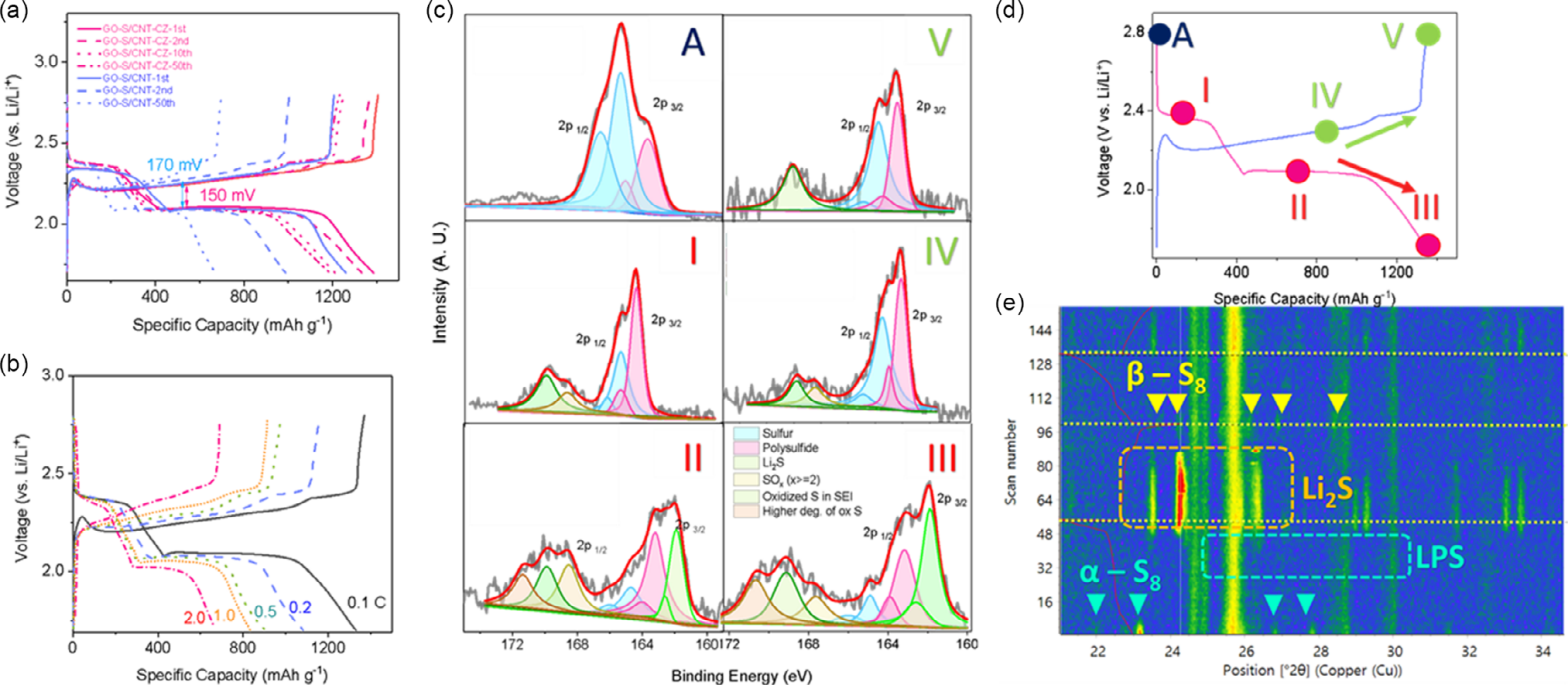
Electrochemical characterization of GO‐S‐CNT‐CZ. a) Discharge/charge profiles of versus GO‐S electrode at various cycles. b) Discharge/charge profiles at various rates. c) Postmortem XPS results at every discharging/charging states defined in (d). d) Definition of various discharged/charged states (A – OCV; red – discharging; green – charging). e) In situ XRD results with respect to charge/discharge in the first two cycles (4 segments, 1st discharge, 1st charge, 2nd discharge and 2nd charge in that order), the scan number (*y*‐axis) is proportional to the acquisition (discharge/charge) time (h).

With the confirmation of the binding host and the catalytic remediation capability of the composite, we further subjected the standard composites (GO‐S‐CNT‐CZ, red) to electrochemical characterization as proof‐of‐concept coin cell device. **Figure** **3**a compares the discharge/charge profiles of standard to the control samples (GO‐S, blue). The overpotential of the GO‐S‐CNT‐CZ exhibits significant improvement over the control samples at 150 versus 170 mV indicating higher activation and initial Coulombic efficiency (CE). The discharge plateaus at 2.4–2.09 and 2.08–1.7 V (versus Li/Li^+^) show very little discrepancies and thus indicate excellent electrochemical stability and low polarization with various cycles. The results are consistent to the cyclic voltammetry (CV) results, as shown in Figure S8, Supporting Information. For the control sample, the redox plateaus display very similar profiles with various cycles and indicating the strong usable potential of active materials in this synthetic form. Both samples’ CV profiles were subjected to further testing at various rates to reveal more electrochemical characteristics which will be covered in later sections. In overall, the discharge/charge profiles (and in various rates, Figure 3b) from the above results exhibit two distinctive cathodic profiles, referred to first as the reduction of S_8_ to Li_2_S_*x*_ (4 ≤ *x* ≤ 8) and subsequently to Li_2_S_2_/Li_2_S as well as a single, strong anodic peak that accounts for a progressive oxidation to sulfur. Even though the results are very similar to reported cases,^[^ ^52^, ^53^ ^]^ the discharge/charge products with respects to various voltage (Figure 3c) were investigated via postmortem X‐ray photoelectron spectroscopy (XPS) analysis to confirm these electrochemical attributes. The discharged products associated with the high voltage discharge plateau exhibit signals that ascribed to long‐chain LPS, mostly indicative by the doublet of 2p_3/2_ centered at around 162.8 eV (Stage I, Figure 3d). Stage II specifies the subsequent low discharge plateaus and related process. Apart from the LPS, the contribution by Li_2_S_2_/Li_2_S (2p_3/2_ centered at 161.6 eV) is more obvious and significant. At stage III, the contribution by the Li_2_S surpasses in intensity to LPS contribution indicating the complete discharged products of Li_2_S. At the anodic reaction, the anodic peak as shown in the CV result is complemented by the long progressive plateaus in the charge profile. At stage IV, the contribution from Li_2_S_2_/Li_2_S progressively diminishes. At stage V (2.8 V versus Li/Li^+^), the charged products are able to return to molecular sulfur in which the doublet associated with S_8_ was identified (163.9 eV, 2p_3/2_). From the findings, we confirm the nature of the discharged products at each cathodic plateaus to Li_2_S_*x*_ (4 ≤ *x* ≤ 8), and Li_2_S_2_/Li_2_S to sulfur, respectively, from high to low voltages. In the anodic reaction, the postmortem XPS analysis provides convincing proof of the charged products and formation of sulfur toward the fully charged state at stage V. These electrochemical signatures at various voltage were further confirmed via operando XRD analysis (Figure 3e). The results confirm with the above postmortem XPS investigation. At stage I, the XRD signals attributed to that of S_8_ are diminishing and weaker due to the conversion of sulfur to the LPS species in the solid–liquid transition.^[^ ^52^ ^]^ The formation of Li_2_S is starting to become more prominent at the second half of stage II toward 1.7 V. At the charging/anodic reaction, the progressive oxidation of the active materials results in the deconstruction of the LPS and Li_2_S species are clearly indicated. However, it is worthwhile to note that the formation of the sulfur species experiences irreversible phase transition from *α*‐S_8_ to *β*‐S_8_ (JCPDS‐071‐0137). The reaction could be a result due to the cycled/activated sulfur incorporation into the CNT structure due to the high adsorption capability of the CoS catalysts, as shown in earlier attempts of stabilizing starting active sulfur using CNT.^[^ ^54^ ^]^ In overall, such attempts have been shown to result in a good cycling performance with the use of a highly conductive CNT.

**Figure 4:**
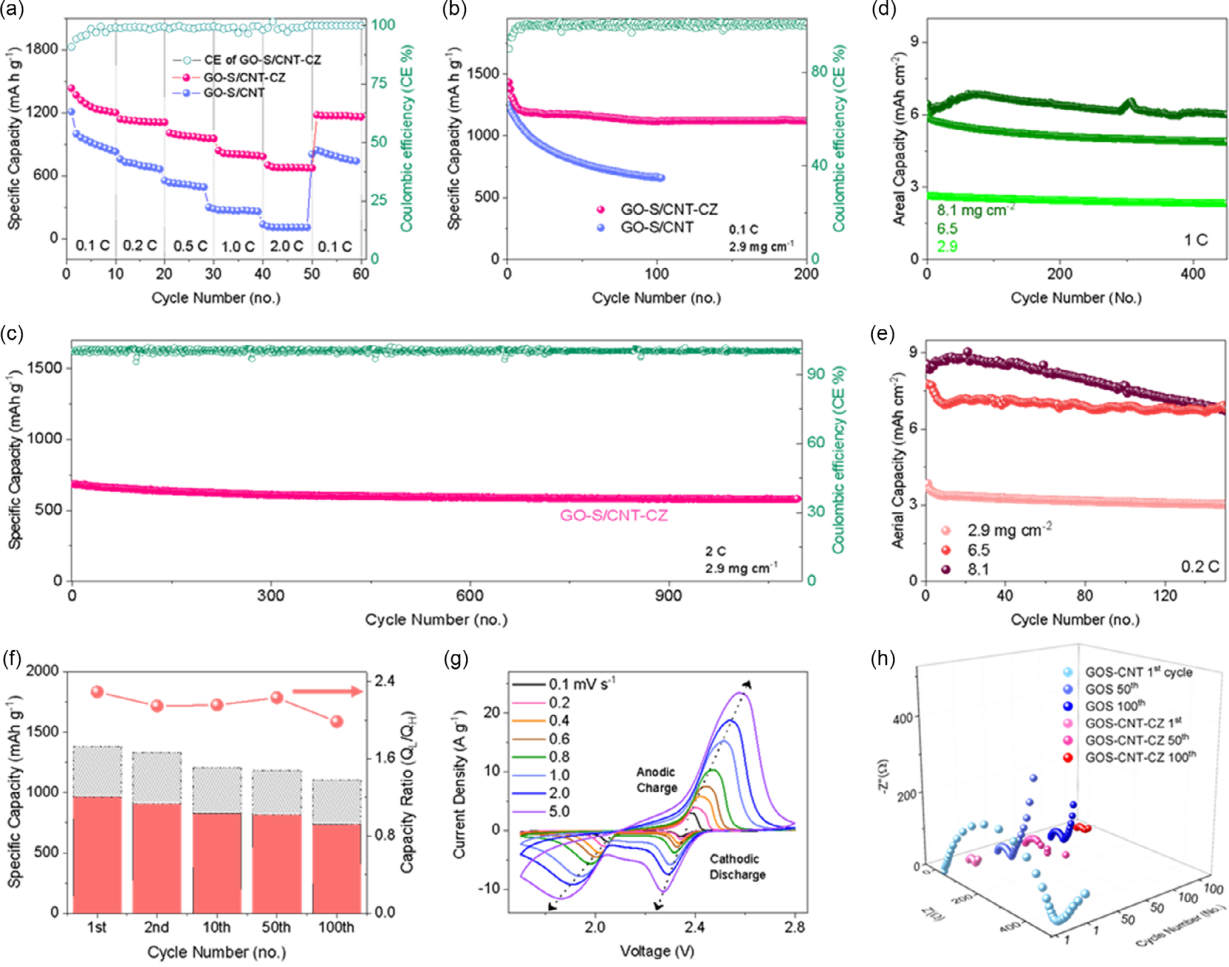
Device performance in comparing GO‐S‐CNT‐CZ versus GO‐S as electrode materials. a) Rate performance and Coulombic efficiency of GO‐S‐CNT‐CZ. b,c) Cycling performance at low rate 0.2 C (b), and high rate 1.0 C (c); d,e) Performance stability of various areal loadings of electrode materials of GO‐S‐CNT‐CZ at low rate 0.2 C (d) and high rate at 1.0 C (e). f) *Q*‐ratio at various cycles of the GO‐S‐CNT‐CZ electrode. g) CV profiles at various scan rates of GO‐S‐CNT‐CZ electrode. h) EIS profiles of both electrodes compared at various cycles.

Both electrode materials were further assessed on its performance (C‐rate and stability performance). Specific capacities of 1220, 1115, 975, 804 and 678 mAh g^−1^ were measured at 0.1, 0.2, 0.5, 1.0, 2.0 C, respectively (1 C = 1675 mA g^−1^), a clear and significant rate performance advantage over the control electrodes at 828, 663, 496, 268, and 106 mAh g^−1^ at identical rates (**Figure** **4**a). The polarization at various rates was also examined (Figure 3a). Apart from the larger overpotentials in higher rates, suggesting kinetic stress in higher rate requirements, profiles at various rates show almost similar features and indicate high electrochemical consistencies at these various rates. Reverting the performance evaluation from 2.0 to 0.1 C shows immediate response, in which the specific capacities for both samples are able to return to their initial recorded level of specific capacities respectively. However, the results show that GO‐S experienced a significant capacity decay. Further cycling stability test at 0.1 C further necessitates the examination (Figure 4b). For the standard electrode, apart from the slight performance drop at the first few cycles as materials activation, the specific capacity (e.g., 20th versus 100th cycle) shows almost no drastic deterioration. This is in stark contrast to the control samples, in which the capacity decay is almost consistent and continued to higher cycles. It is evident that without catalytic support, the active materials do not display adequate performance metrics for realistic application and this result embodies the importance of CoS catalytic support in realizing applicable performance for this materials construct and approach.

To further examine the performance stability of the composite GO‐S‐CNT‐CZ, we subjected the electrode devices to stress test the cycling performance under high rate (2.0 C). At >1800th cycle (Figure 4c), the specific capacity shows very high‐performance consistency with low‐capacity decay (<2.8%, CE at >99.9%). To also examine the effect of areal mass density of the electrode, a critical factor for practical application, the stability testing was also further conducted on standard electrode with thicker loadings (8.1, 6.5 mg cm^−2^) and were compared to 2.9 mg cm^−2^ (default settings). All the electrode materials were manufactured using the same inventory and process protocols, with the only differing factor is by the areal loadings. First at high rate (1.0 C), we established that the cycling stability of all three samples follows the same trend and exhibits excellent performance stability (>450 cycles). The overall trend (Figure 4d) exhibits proportional areal capacity versus loadings (6.0, 4.9, and 2.35 mAh cm^−2^, 450th cycle), and low degradation (11.0, 10.0, 9.2% with areal loadings). The same trend is also observed (Figure 4e) when the same batch of samples was assessed at low‐rate cycling (0.2 C), in which excellent performance stability is displayed (>150 cycles). It is worthwhile to point out that at high loading of 6.5 mg cm^−2^, the high areal capacity of 6.4 and 4.9 mAh cm^−2^ are retained at higher cycles and demonstrate excellent performance (rate and performance stability) of the construct in attributing to high‐capacity utility (Figure S9, Supporting Information). In other words, the excellent rate and cycling stability exhibit consistent performance for large areal capacity and these results are highly suggestive of upscaling prospects of these synthetically derived active materials construct.

To further investigate the superior performance characteristics of the standard composite, the high‐ and low‐voltage discharged specific capacities (denoted as *Q*_H_ and *Q*_L_) were subject to comparison at various rates to demonstrate the efficacy of catalytic support. The ratio of low‐to‐high‐capacity ratio (*Q*‐ratio = *Q*_L_/*Q*_H_) is reflecting on the ability of the catalytic support in catalyzing the discharge reaction kinetics. For the catalytically support sample (GO‐S‐CNT‐CZ), high *Q*‐ratio were displayed at 2.10, 2.05, 1.97, 1.63, and 1.46 with respect to various rates indicating consistent catalytic performance (Figure S10a, Supporting Information). The higher capacities at low voltage plateau represent effectiveness of the discharging process ascribed to its excellent catalytic ability. The decreasing *Q*‐ratio with increasing rates indicates that it is vital for catalytic support in delivering commendable electrochemical kinetics, which is especially necessary at higher rates. The performance stability of the catalytic support was also investigated at various cycles (Figure 4f). We established that the catalytic support at various cycles retains very high electrochemical stability (*Q*‐ratio ≈ 2.0, from 1st to 100th cycle). In comparison, the kinetic metrics fares better than that of the control sample (Figure S10b, Supporting Information) in which a substantial difference and lower *Q*‐ratio is observed (GO‐S, *Q*‐ratio ≈ 1.50). The higher *Q*‐ratio of standard versus control samples signifies that the catalytically support electrode materials are more capable to effectively catalyzing LPS to solid Li_2_S. The consistent values throughout various cycles embody consistent kinetic properties at various cycles for both samples. To further validate this electrochemical/kinetic characteristic, electrochemical impedance spectroscopy (EIS) of both standard and control samples was investigated at various rates and cycles (1st, 10th, 50th, and 100th cycle). The typical EIS result records two trajectories of half circles of increasing radius, corresponds to the high‐ and low‐frequency profiles, respectively. We are particularly interested in the intersection point between the two semicircles, in which is proportional to the charge‐transfer impedance (*R*_ct_) representing that of the kinetic properties of the discharge/charge process. The result of EIS of standard samples at various cycle (Figure 4h) exhibits almost constant profile, a testament that stability of performance and ability of the catalytic support persisting to higher cycles and a low impedance profile (Figure S11a, Supporting Information), signifying faster ionic diffusion and LPS conversion kinetics.^[^ ^55^ ^]^ In stark contrast, EIS results of control samples (Figure S11b, Supporting Information) at the same cycles exhibit considerable larger impedance and thus with less desirable electrochemical kinetic metrics. In addition, we further investigated the diffusion kinetics and subjecting standard electrode to CV testing via increasing scan rates (*υ*, mV s^−1^), as previously mentioned (Figure 4g). First, the CV profiles at various scan rates remained consistent with the previous presented result at 0.1 mV s^−1^. The CV profiles of the control samples were also evaluated with respect to various cycles (Figure S12, Supporting Information). For both electrode materials, we are particular interested in the relationship of the fitted peak currents (*I*_P_) of all the reactions and the relationship to the square roots of scan rates via classical Randles–Sevcik equation, in which *I*_P_ ∝ *υ* ^1/2^.^[^ ^56^, ^57^ ^]^ From the Randles–Sevcik equation, the ion diffusion coefficient (*D*) is proportional and can be evaluated by the slope of the linear regression of relationship of the above two variables (Figure S13, Supporting Information).^[^ ^58^ ^]^ In stark contrast to the control samples, the gradient is substantially higher for the standard samples. As a conclusion, this provides another convincing proof on the catalytic support in empowering diffusion kinetics as effective Li–S battery material.

## 3 Conclusion

Substantial proofs demonstrated and supported the performance prospects of the unique synthetically derived active materials/catalytic composites via the use of low dimensional carbonaceous materials with catalytic support. The large active material loading was investigated by larger areal loading applications (6.9 and 8.1 mg cm^−2^) and exhibits stable performance metrics both in low and high rates. Contrasting recent works on achieving high active materials loading, we established and concluded that this materials design approach and potential scalable attributes are able to provide large active materials loading without compromising performance stability at various rates (0.2 to 1.0 C). (Table S1, Supporting Information). To elicit excellent performance, it is necessary to subject active materials to electrocatalytic support. The CoS‐based electrocatalyst not only provides high adsorption and LPS remediation capability, but also the efficacy in working synergistically via the low‐dimensional carbonaceous construct (with rGO in tandem with CNT) with synthetically derived sulfur is also evident in providing stable performance at various rates due to the high adsorption of CoS‐based catalysts working together with CNT (high surface area CNT‐CZ) in containing the charged/discharged products. These results provide further justification of the superior performance of the composite construct and the effectiveness of the catalytic support in engaging activation and proper utilization of the synthetically derived active materials. With the high performance and straightforwardness in processing fabrications, as well as adaptation prospects in various practical attributes, we demonstrated and presented an attractive approach as a prospective starting point for practical manufacturing adaptations and is vital in today's requirement in realizing both high performance and practical upscaling potentials.

## 4 Experimental Section

4.1

4.1.1

##### Synthesis of GO‐S

The synthesis of GO follows from our previous works^[^ ^51^ ^]^ and is controlled to 4 mg mL^−1^. 4.5 mL of the GO solution was added with 320 mg of sodium persulfate (Na_2_S_2_O_8_). 2.0 mL of 2 m hydrochloric acid (97% v/v, HCl) was then added slowly and dropwise to the composite solution under vigorous stirring (>120 min) at room temperature. 0.2 mL of 1 m ascorbic acid was then added to the solution and heated at 80 °C for 4 h. After cooling down, the solute was thoroughly washed with Milli‐Q water, freeze dried, and collected as GO‐S composite material.

##### Synthesis of CNT‐CZ

Four separate solutions were prepared as indicated (solution A, B, C, D). 200 mL of methanol was added with 1 g of polyvinylpyrrolidone (PVP, 40 000 MW) and stirred until fully dissolved as solution A. 200 mg of CNT (XFNano) was added to 25 mL of methanol under stirring and as solution B. 498 and 240 mg of zinc and cobalt nitrate hexahydrate (Zn(NO_3_)_2_ · 6H_2_O; Co(NO_3_)_2_ · 6H_2_O), respectively, were dissolved by rapid stirring in 40 mL of methanol as solution C. 1.7 g of 2‐methylimidazolate was fully dissolved in 25 mL of methanol via rapid stirring as solution D. As a second step, solution B was added dropwise to solution A under sonication, which was contained at iced bath conditions (around 0 °C) for 45 min. Subsequently, solution C was added slowly and dropwise to the precursor solution, following that with solution D in a similar fashion, and proceeded with sonication for another 30 min. The resultant precursor solution was then transferred to magnetic stirring for 120 min, and then left for aging >24 h. After the aging process, the solute was collected and washed via centrifuge method with methanol. Finally, the solute was freeze dried and collected as CNT‐ZIF. To synthesize CNT‐CZ, a required amount of synthesized CNT‐ZIF was placed at a ceramic boat with sulfurizing precursor (sodium hydrosulfide, NaSH), with the later at the upstream location. The boat loaded with both precursors was then transferred to a tube furnace and sealed within argon‐filled environment. The sulfurization/pyrolysis commences at 350 °C for 120 min and then proceeds onto 500 °C for 480 min. The resultant product (CNT‐CZ) was then collected after the tube furnace cooled down naturally.

##### Synthesis of GO‐S‐CNT‐CZ

5.0 mL of as‐prepared GO‐S solution (20 mg) and 5.0 mL of CNT‐CZ (10 mg) were subject to sonication in iced bath environment for 30 min. Subsequently, the CNT composite solution was added dropwise and gradually to the GO‐S solution while under iced sonication. The resultant solution was to remain sonicated for another 45 min. After the sonication process and within proper containment, the solution was then transferred into a liquid nitrogen environment which it was then rapidly frozen and remained for a couple of minutes. Then the solution was transferred for freeze drying process for >72 h and collected as GO‐S‐CNT‐CZ. For the synthesis of GO‐S‐CNT, the synthesis process was identical in which CNT‐CZ is replaced by pristine CNT.

##### Synthesis of Li_2_S_6_ and Adsorption Test

The synthesis of Li_2_S_6_ solution follows our previous work.^[^ ^16^, ^17^ ^]^ In essence, Li_2_S and sulfur in a molar ratio of 1:5 was mixed as solute and added with 1,3‐dioxolane/1,2‐dimethoxyethane (DOL/DME) as solvent in 1:1 v/v. The resulting solution was stirred overnight under magnetic stirring at 60 °C resulting in the formation of Li_2_S_6_ solution. To proceed with the adsorption test, 10 mg of test subjects (CNT‐CZ or CNT) were added with 4 mL of the above as prepared solution. The mixtures were allowed to rest for 6 h before observation and quantitative measurement by UV–visible spectroscopy.

##### Materials Characterization

The morphologies and microstructure of the materials constructs were investigated by field‐emission scanning electron microscope (FESEM, JEOL‐7600F), with energy dispersive X‐ray spectroscopy (EDS, Oxford instruments) capability, and high‐resolution transmission electron microscopy (HR‐TEM, JEOL‐2011). X‐ray diffraction (XRD, Bruker D8 Advance) was conducted on Cu Kα (*λ* = 0.154 nm) radiation. N_2_ adsorption/desorption isotherms were measured to investigate and quantify surface area, the porosity and distribution profiles via the automated gas sorption analyzer (Autolab‐iQ, Quantachrome Instruments). The specific surface area was evaluated via BET method. The porosity profiles were evaluated via Barrett–Joyner–Halenda (BJH) method. X‐ray photoelectron spectroscopy (XPS, PHI 5600) was conducted on Al Kα X‐ray source (350 W, pass energy 29 eV). Thermogravimetric analysis (TGA) was conducted in required temperature range.

##### Calculations of Gibbs Free Energy and Adsorption Energy

Based on classical thermodynamics at room temperature, the Gibbs free energy of adsorption governs the process instead of the adsorption enthalpy^[^ ^59^ ^]^

(1) $$\Delta G\left(\text{ads}\right)=G\left({\text{Li}}_{2}S_{n}\text{/Slab}\right)–G\left({\text{Li}}_{2}S_{n}\right)–G\left(\text{Slab}\right)$$

where Δ*G*(ads), *G*(Li_2_S_*n*_)/Slab), *G*(Li_2_S_*n*_), and *G*(Slab) are the Gibbs free energy of the CoS or graphitic carbon slabs after the adsorption of Li_2_S_*n*_, the Li_2_S_*n*_ systems, and the CoS or graphitic carbon slabs, respectively. The Gibbs free energy of adsorption has both the enthalpy and the entropy contributions, which is calculated by the relation of Δ*G*(ads) = Δ*H*(ads) − *T*Δ*S*(ads). The binding energy *E*_b_ is defined as

(2) $$E_{b}=E\left({\text{Li}}_{2}S_{n}\text{/Slab}\right)\;–E\left({\text{Li}}_{2}S_{n}\right)\;–E\left(\text{Slab}\right)$$

where *E*(Li_2_S_*n*_/Slab), *E*(Li_2_S_*n*_), and *E*(Slab) are the total energies of the CoS or graphitic carbon slabs after the adsorption of the LPS (Li_2_S_*n*_), the LPS systems, and the CoS or graphitic carbon slabs.

##### DFT Computational Methods

DFT calculations for the electronic energies were performed using the Vienna ab initio software package (VASP).^[^ ^60^, ^61^ ^]^ The generalized gradient approximation (GGA)^[^ ^62^ ^]^ as formulated by Perdew–Burke–Ernzerhof (PBE)^[^ ^63^ ^]^ functional was adopted for the exchange‐correlation potential. The cutoff energy is chosen as 500 eV and Brillouin zone was sampled using a 5 × 5 × 1 Γ‐centered *k* mesh. The van der Waals (vdW) interaction was included using the DFT‐D3 method.^[^ ^64^ ^]^ For geometry optimization, the convergence criteria for energy and force were set as 10^−4^ eV and 0.01 eVÅ^−1^, respectively. The expanded 4 × 4 supercell of graphitic carbon and a 3 × 2 supercell of CoS slab were utilized. A vacuum spacing of at least 20 Å was included to avoid the interaction between the subsequent and concurrent slabs. The dipole moment corrections are adopted to check the effect of charges. The VASPKIT was utilized to perform the adsorption model free energy correction.^[^ ^65^ ^]^ LiS_*n*_ free energies correction was obtained by computing the phonon dispersion correlations and combined with PHONOPY software methods.^[^ ^66^ ^]^

##### Electrochemical Device Assembly, Characterization, and Performance Evaluation

Standard (GO‐S‐CNT‐CZ) and control samples (GO‐S) were used as cathode materials and prepared with the following protocols as proof‐of‐concept coin cell device (CR2032). The cathodes were prepared, specifically, by mixing of the standard/control composite samples with conductive carbon black (Ketjenblack) and with polyvinylidene fluoride binder (PVDF) in 8:1:1 w/w with *N*‐methylpyrrolidone (NMP) as solvent. The resultant solution slurry was then coated onto aluminum foil (current collector and support) and dried at 60 °C > 12 h via the use of vacuum oven to process into cathode materials. The coated electrodes were then punched into perfect circular shapes with diameter of 1.2 cm. Following the same process as mentioned, various thickness exemplifying areal loadings (2.9, 6.5, 8.1 mg cm^−2^) of electrode materials were prepared by varying the coating areas. Pristine lithium metal foils were used as counter electrode and tailored to the same size as the prepared electrodes. To prepare the electrolyte, 1 m of lithium bis(trifluoromethanesulfonyl)imide (LiTFSI) in DOL/DME (1:1, v/v) was utilized with the supplementation of 0.2 m lithium nitrate (LiNO_3_) as additive. The assembly of the coin cells were carried out in argon‐filled and controlled environment with moisture and oxygen level kept well below 0.5 ppm. All coin cell devices were assembled with strict protocols in which the electrolyte/sulfur ratio is kept at 10 μL mg^−1^, and with the use of Celgard PP membrane as separator. During the assembly, all the components of the assembly including the electrodes and separator were kept in perfect alignment. CV and EIS electrochemical characterizations were carried out on a VMP3 Biologic potentiostat. Galvanostatic performance discharge/charge tests in various rates and cycling were carried out on a multichannel Neware testing facility (battery tester) in the range of 1.7–2.8 V versus Li/Li^+^ at constant current mode.

## Conflict of Interest

The authors declare no conflict of interest.

## Supporting information

Supplementary Material

## Data Availability

The data that support the findings of this study are available from the corresponding author upon reasonable request.
