# Supplementary material for: In Situ Synthesis Method of Approaching High Surface Capacity Sulfur and the Role of Cobalt Sulfide as Lithium–Sulfur Battery Materials
Source: Small Sci. 2023 Aug 17;3(10):2300070. doi: 10.1002/smsc.202300070 (PMC11935898; doi:10.1002/smsc.202300070)
Supplement: Supplementary file 1 — Supplementary Material [file SMSC-3-2300070-s001.pdf]

Supporting Information

In Situ Synthesis Method of Approaching High Surface Capacity Sulfur and the Role of Cobalt Sulfide as Lithium–Sulfur Battery Materials

Yew Von Lim,[1] Sareh Vafakhah,[3] Xueliang Li,[1] Daliang Fang,[1] Shaozhuan Huang,[4] Ye Wang,[5] Yee Sin Ang,[2] Lay Kee Ang,[2] Hui Ying Yang[1†]

[1]Pillar of Engineering Product Development, Singapore University of Technology and Design, 8 Somapah Road, Singapore 487372

[2]Pillar of Science, Mathematics and Technology, Singapore University of Technology and Design, 8 Somapah Road, Singapore 487372

[3]School of Chemical and Biomolecular Engineering, the University of Sydney, Darlington NSW, Australia 2006

[4]Key Laboratory of Catalysis and Energy Materials Chemistry of Ministry of Education, South-Central University of Nationalities, Wuhan, Hebei 430074, PR China

[5]Key Laboratory of Materials Physics, Ministry of Education, School of Physics and Microelectronics, Zhengzhou University 450052, PR China

[†]*Corresponding author:* *yanghuiying@sutd.edu.sg*

Results

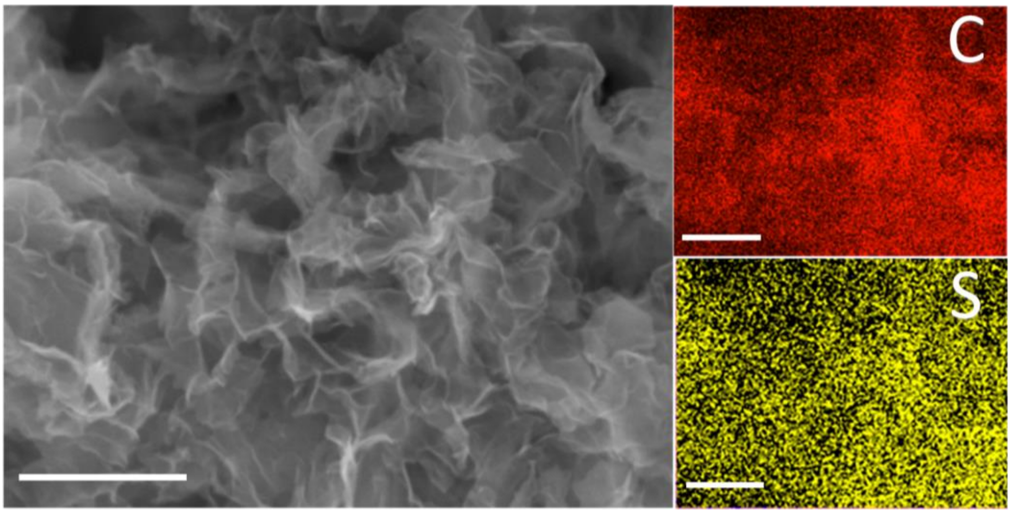

Figure S1. SEM and corresponding EDS results of the GO-S composite precursor. (scale bar at 1 μm).

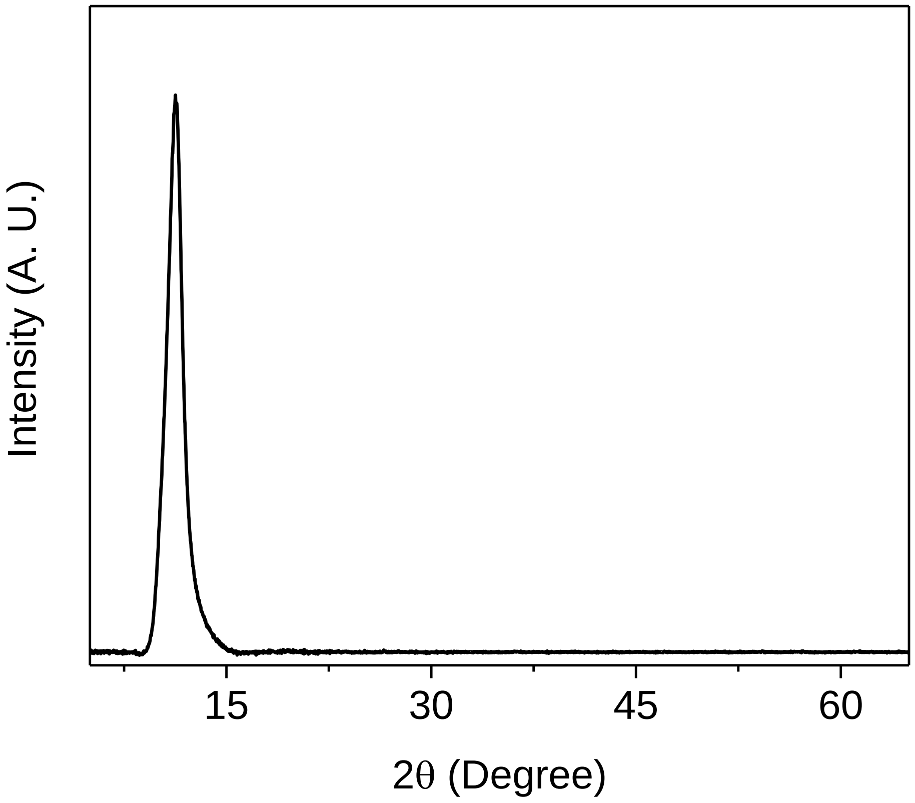

Figure S2. The XRD result of the GO without reduction treatment.

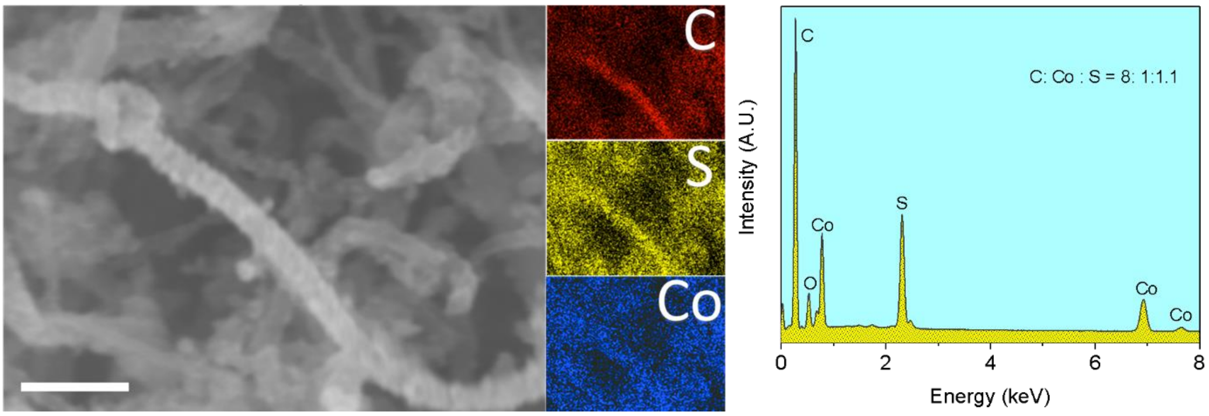

Figure S3. Morphology of the CNT-CZ in the SEM results and the corresponding EDS mapping images. (Scale bar = 500 nm.)

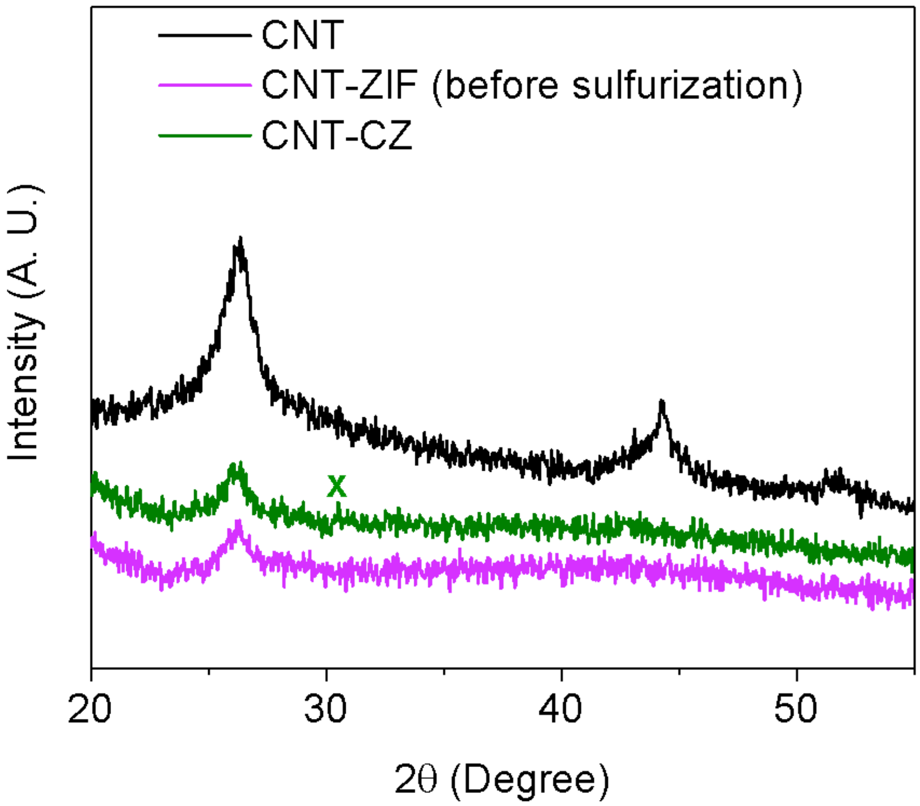

Figure S4. XRD pattern of various CNT host precursors. The indicator × represents the formation of CoS (100) XRD pattern.

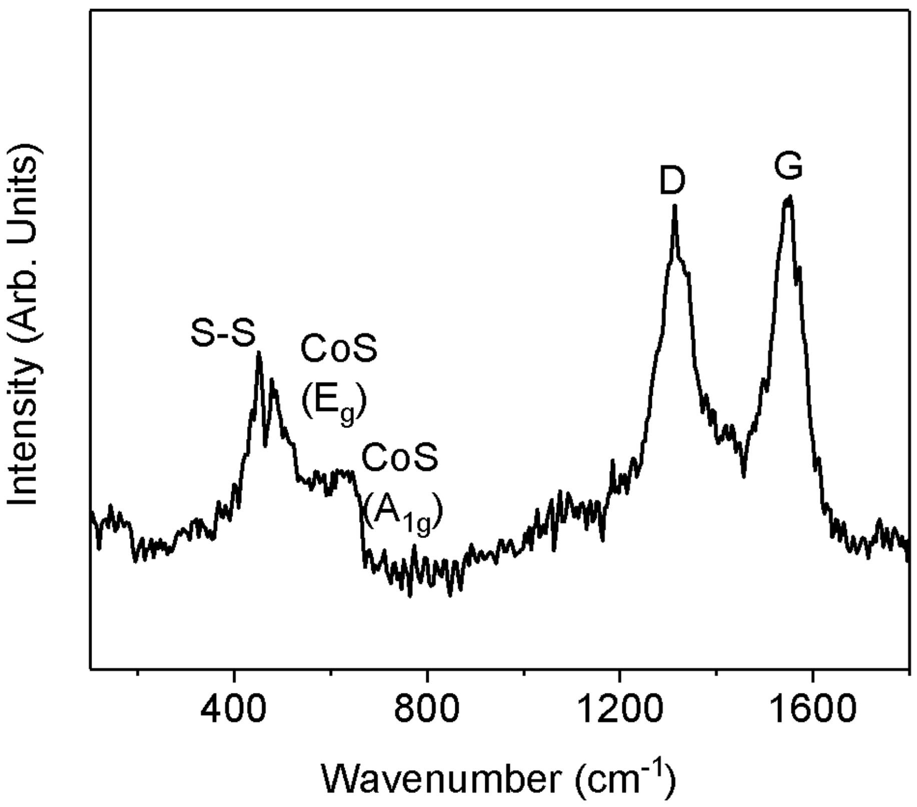

Figure S5. Raman spectra and results of the GO-S-CNT-CZ composite. The significant D and G band indicates the formation of the graphitic carbon and the success of the reduction process to rGO.

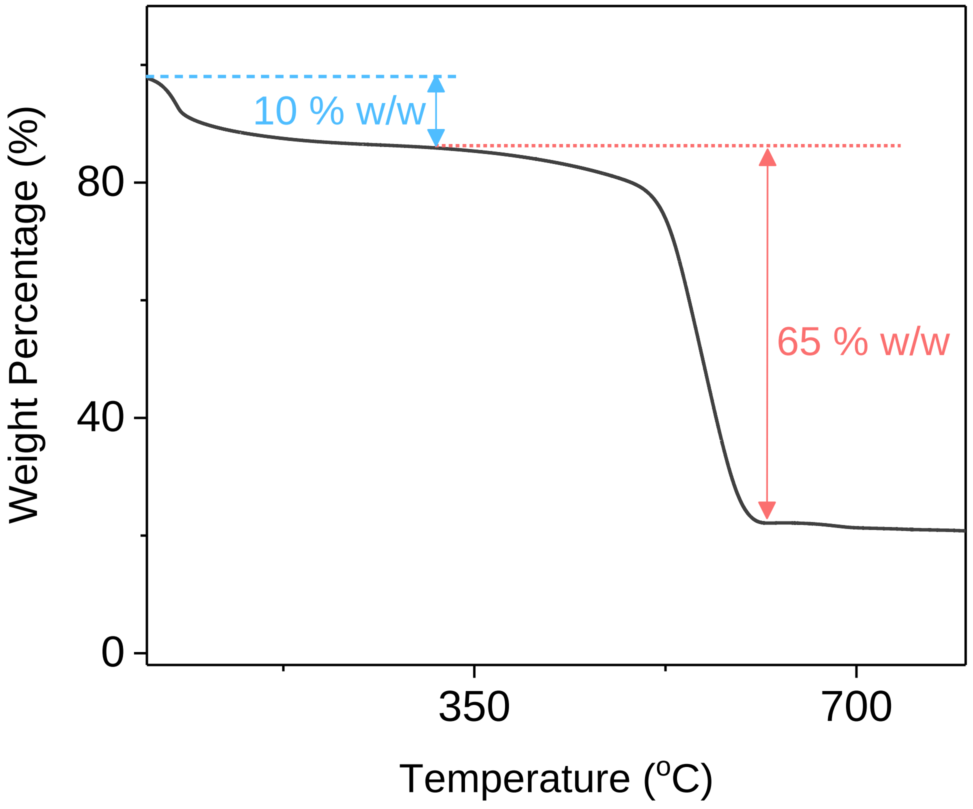

Figure S6. TGA results of the CNT-CZ composite precursor, operated from 30 – 750 °.

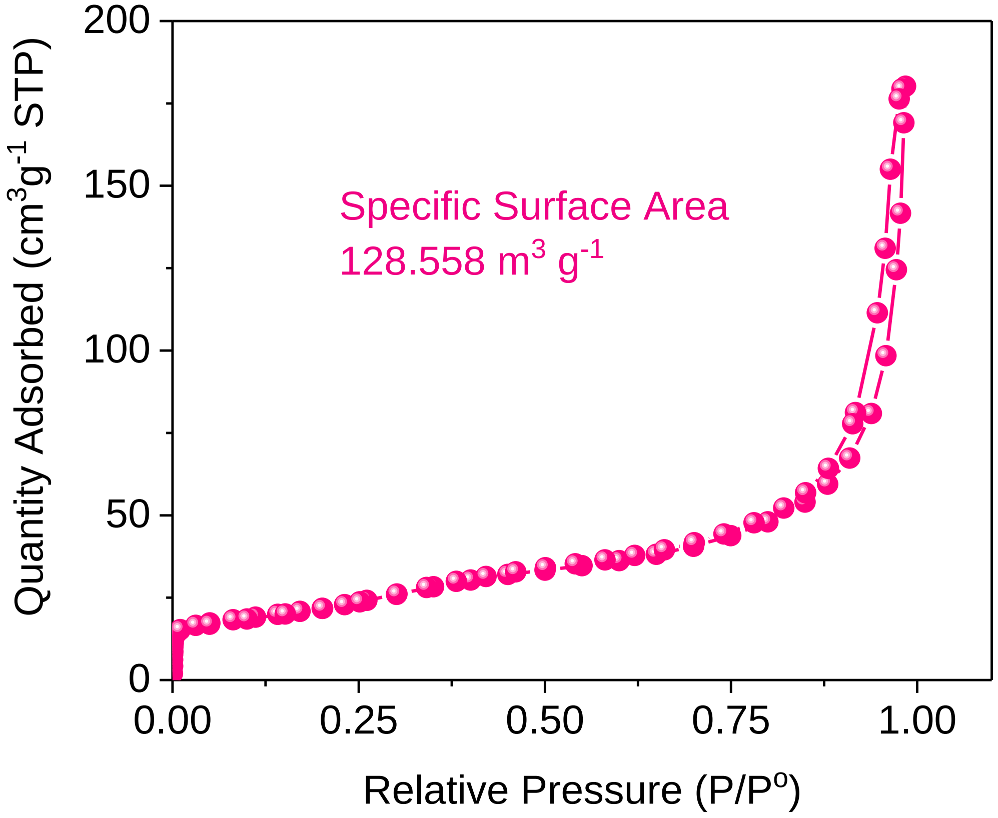

Figure S7. N_2 sorption isotherm of the CNT-CZ catalyst precursor. The specific surface area is evaluated by the Brunauer-Emmett-Teller (BET) method.

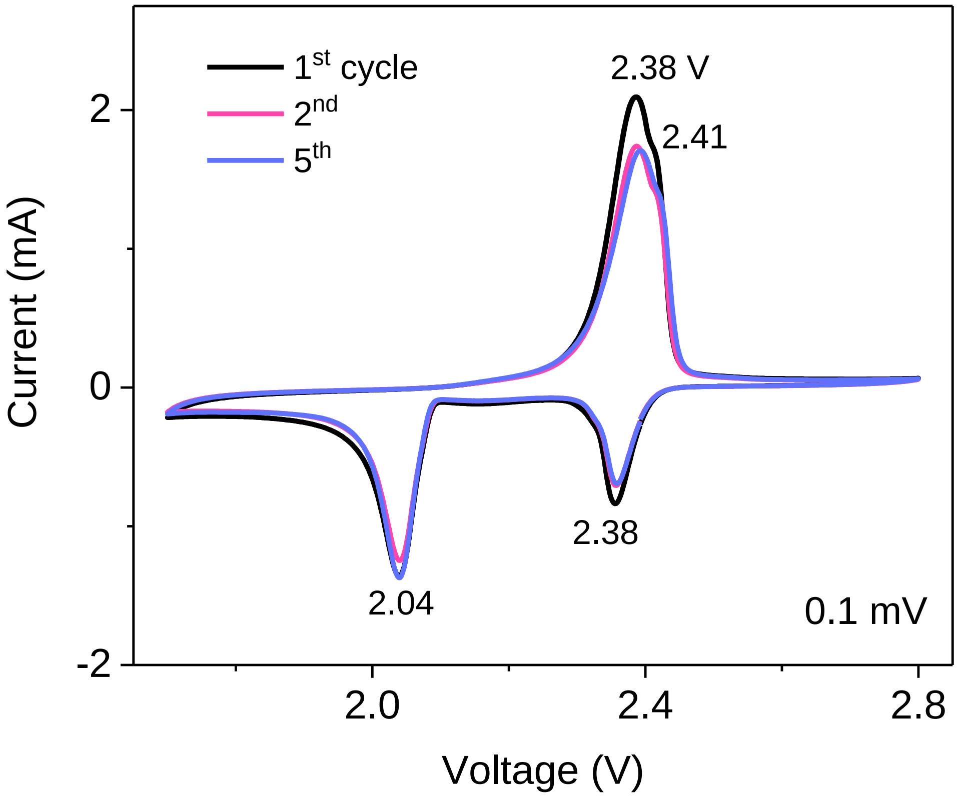

Figure S8. Cyclic voltammetry (CV) results of GO-S-CNT-CZ conducted at 0.1 mV s^{-1} at various cycles.

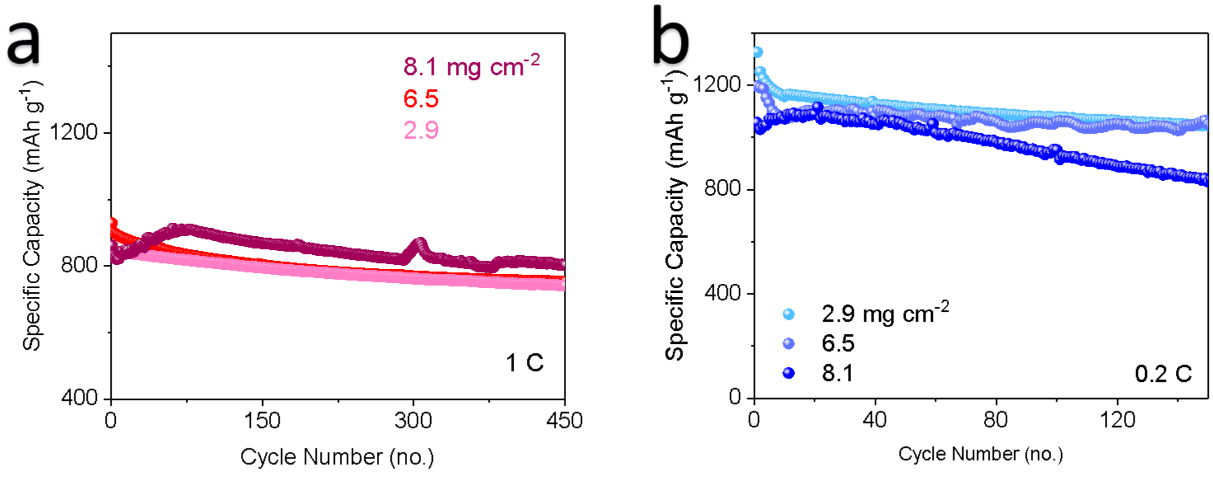

Figure S9. Cycling stability in terms of the specific capacities (mAh g^{-1}) of various areal loadings at (a) high rate discharge/charge 1.0 C, and (b) low rate discharge/charge 0.2 C.

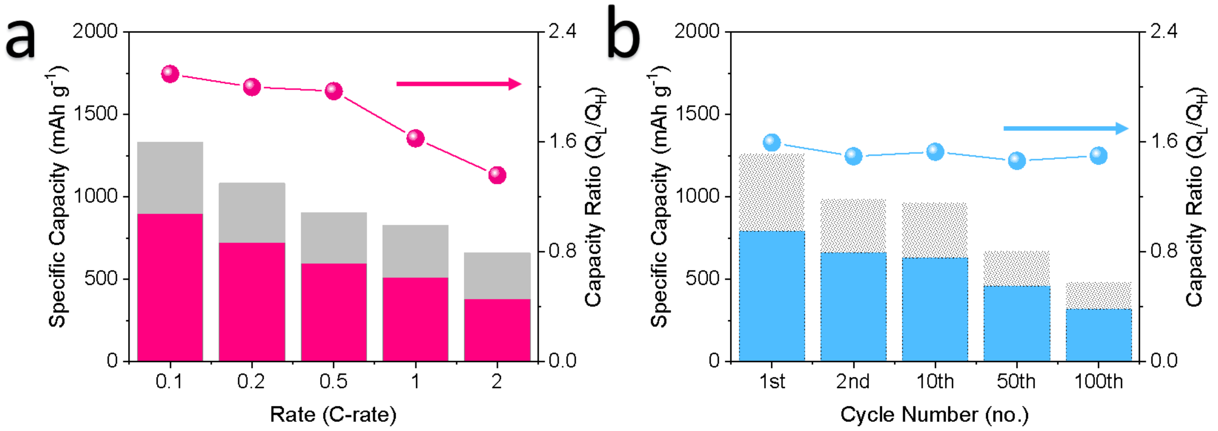

Figure S10. The Q-ratio results: (a) at various discharge/charge rates of GO-S-CNT-CZ; (b) at various cycles of GO-S electrode materials.

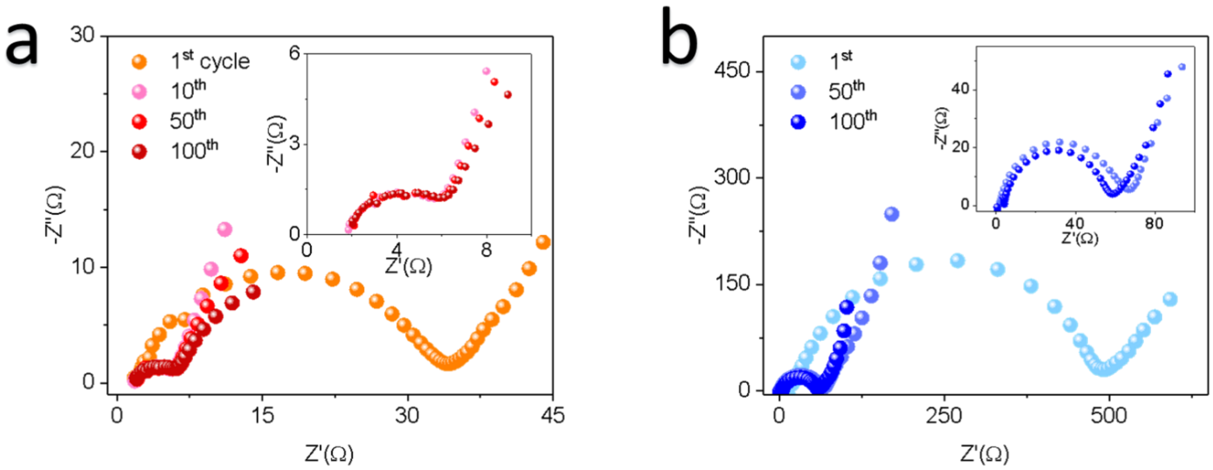

Figure S11. EIS results at various cycles of: (a) GO-S-CNT-CZ, and (b) GO-S electrode materials.

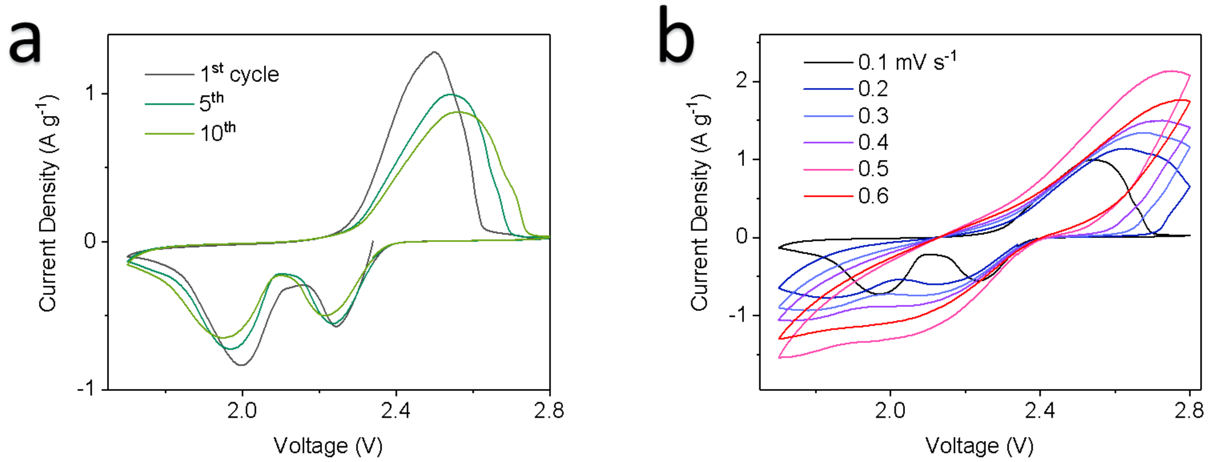

Figure S12. CV results of GO-S electrode materials at: (a) various cycles; (b) various scan rates.

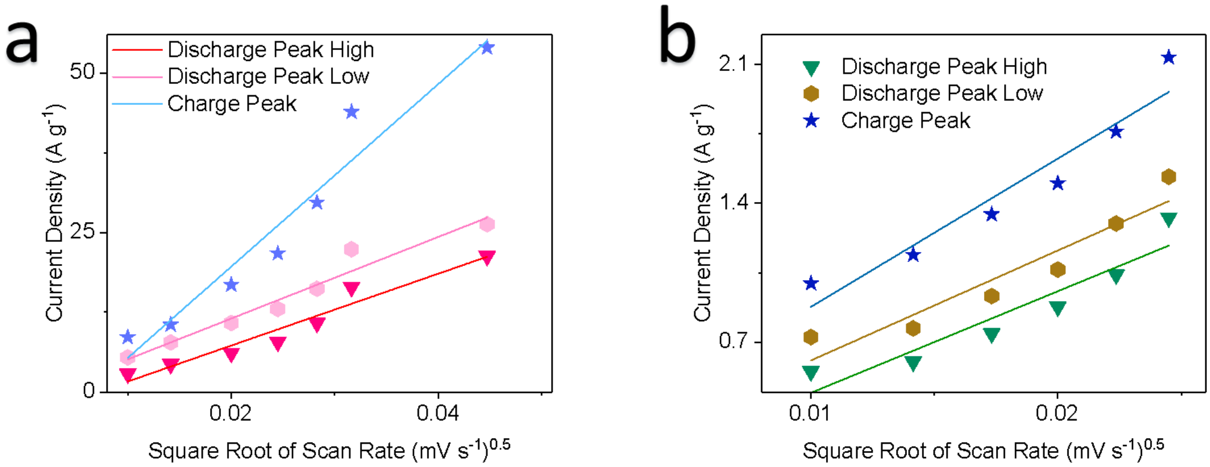

Figure S13. The linear regression of the current density vs. square root of the scan rates from the three redox reactions (high voltage discharge, low voltage discharge, and the charging reactions) of: (a) GO-S-CNT-CZ, and (b) GO-S electrodes.

Table S1. Recent works contrasting this work on performance and active materials loading

Samples	Cycling Performance	Active Materials Loading (mg cm^{-2})	Cathode/Sulfur	Ref
NCN-S	0.2 C/1309 mAh g^{-1}/200 cycles/797 mAh g^{-1}	5	1.68	[1]
3D-PG/S	1.5 mAcm^{-2}/1003 mAhg^{-1}/400 cycles/714 mAh g^{-1}	4	1.72	[2]
Li_2S_6/C	0.1 C/912 mAh g^{-1}/50 cycles/702 mAh g^{-1}	61.4	-	[3]
POMS/S	0.2 C/5.7 mAhcm^{-2}/100 cycles/4.4 mAh cm^{-2}	6.5	1.67	[4]
GF-rGO/S	0.2 C/1000 mAh g^{-1}/350 cycles/645 mAh g^{-1}	9.8	-	[5]
S/N-IOP	0.1 C/1309 mAh g^{-1}/150 cycles/734 mAh g^{-1}	6	1.56	[6]
CMP/S	0.5 C/-/200 cycles/6.35 mAh cm^{-2}	10.9	1.54	[7]
ePCNTM/S	0.1 C/-/50 cycles/3.2 mAh cm^{-2}	6.5	1.98	[8]
S@P/MCB	1.0 C/5.5 mAhcm^{-2}/150 cycles/-	5.02	1.78	[9]
S-PCNTs	0.2 C/1097 mAh g^{-1}/100 cycles/976 mAh g^{-1}	4.6	1.67	[10]
S@PCS	0.2 C/3.5 mAhcm^{-2}/50 cycles/4.8 mAh cm^{-2}	5.5	1.39	[11]
GOS-CNT-CZ	1.0 C/6.0 mAhcm^{-2}/450 cycles/5.35 mAh cm^{-2}<br>1.0 C/4.9 mAhcm^{-2}/450 cycles/4.40 mAh cm^{-2}	8.1<br>6.5	1.25	This work

References

[1] C. Hu, C. Kirk, Q. Cai, C. Cuadrado-Collados, J. Silvestre-Albero, F. Rodríguez-Reinoso, M. J. Biggs, *Adv. Energy Mater.* **2017**, *7*, 1701082.

[2] D. Cheng, P. Wu, J. Wang, X. Tang, T. An, H. Zhou, D. Zhang, T. Fan, *Carbon N. Y.* **2019**, *143*, 869.

[3] S.-H. Chung, C.-H. Chang, A. Manthiram, *ACS Nano* **2016**, *10*, 10462.

[4] B. Lee, T. Kang, H. Lee, J. S. Samdani, Y. Jung, C. Zhang, Z. Yu, G. Xu, L. Cheng, S. Byun, Y. M. Lee, K. Amine, J. Yu, *Adv. Energy Mater.* **2020**, *10*, 1903934.

[5] G. Hu, C. Xu, Z. Sun, S. Wang, H.-M. Cheng, F. Li, W. Ren, *Adv. Mater.* **2016**, *28*, 1603.

[6] P. Han, S. Chung, A. Manthiram, *Small* **2019**, *15*, 1900690.

[7] X. Yu, J. Deng, R. Lv, Z.-H. Huang, B. Li, F. Kang, *Energy Storage Mater.* **2019**, *20*, 14.

[8] Y. Zhang, G. Li, J. Wang, D. Luo, Z. Sun, Y. Zhao, A. Yu, X. Wang, Z. Chen, *Adv. Energy Mater.* **2021**, *11*, 2100497.

[9] Z. Wu, L. Yuan, Q. Han, Y. Lan, Y. Zhou, X. Jiang, X. Ouyang, J. Zhu, X. Wang, Y. Fu, *J. Power Sources* **2020**, *450*, 227658.

[10] J. S. Lee, J. Jun, J. Jang, A. Manthiram, *Small* **2017**, *13*, 1602984.

[11] G. Li, W. Lei, D. Luo, Y.-P. Deng, D. Wang, Z. Chen, *Adv. Energy Mater.* **2018**, *8*, 1702381.
